# Microbial Biosurfactant: *Candida bombicola* as a Potential Remediator of Environments Contaminated by Heavy Metals

**DOI:** 10.3390/microorganisms11112772

**Published:** 2023-11-15

**Authors:** Renata Raianny da Silva, Júlio C. V. Santos, Hugo M. Meira, Sérgio M. Almeida, Leonie A. Sarubbo, Juliana M. Luna

**Affiliations:** 1 Northeast Biotechnology Network (Renorbio), Federal Rural University of Pernambuco, Rua Dom Manuel de Medeiros, Recife 52171-900, PE, Brazil; renatabiology2015@gmail.com; 2 Environmental Process Development (PPGDPA), Catholic University of Pernambuco, Rua do Príncipe, n. 526, Boa Vista, Recife 50050-900, PE, Brazil; juliovasconcelos05@gmail.com; 3 Advanced Institute of Technology and Innovation (IATI), Rua Potira de Brito, n.216, Boa Vista, Recife 50050-900, PE, Brazil; 4 School of Health and Life Sciences, Catholic University of Pernambuco, Rua do Príncipe, n. 526, Recife 50050-900, PE, Brazil; sergio.almeida@unicap.br; 5 Icam Tech School, Catholic University of Pernambuco, Rua do Príncipe, n. 526, Boa Vista, Recife 50050-900, PE, Brazil

**Keywords:** *Candida bombicola*, biosurfactant, heavy metals, bioremediation

## Abstract

Industrial interest in surfactants of microbial origin has intensified recently due to the characteristics of these compounds, such as biodegradability and reduced toxicity, and their efficiency in removing heavy metals and hydrophobic organic compounds from soils and waters. The aim of this study was to produce a biosurfactant using *Candida bombicola* URM 3712 in a low-cost medium containing 5.0% molasses, 3.0% corn steep liquor and 2.5% residual frying oil for 144 h at 200 rmp. Measurements of engine oil tension and emulsification were made under extreme conditions of temperature (0 °C, 5 °C, 70 °C, 100 °C and 120 °C), pH (2–12) and NaCl concentrations (2–12), demonstrating the stability of the biosurfactant. The isolated biosurfactant was characterized as an anionic molecule with the ability to reduce the surface tension of water from 72 to 29 mN/m, with a critical micellar concentration of 0.5%. The biosurfactant had no toxic effect on vegetable seeds or on *Eisenia fetida* as a bioindicator. Applications in the removal of heavy metals from contaminated soils under dynamic conditions demonstrated the potential of the crude and isolated biosurfactant in the removal of Fe, Zn and Pb with percentages between 70 and 88%, with the highest removal of Pb being 48%. The highest percentage of removal was obtained using the cell-free metabolic liquid, which was able to remove 48, 71 and 88% of lead, zinc and iron from the soil, respectively. Tests in packed columns also confirmed the biosurfactant’s ability to remove Fe, Zn and Pb between 40 and 65%. The removal kinetics demonstrated an increasing percentage, reaching removal of 50, 70 and 85% for Pb, Zn and Fe, respectively, reaching a greater removal efficiency at the end of 24 h. The biosurfactant was also able to significantly reduce the electrical conductivity of solutions containing heavy metals. The biosurfactant produced by *Candida bombicola* has potential as an adjuvant in industrial processes for remediating soils and effluents polluted by inorganic contaminants.

## 1. Introduction

Biosurfactants are amphipathic molecules with both hydrophilic and hydrophobic portions, which are divided between phases with different polarities (oil/water and air/water), causing a decrease in surface tension and increasing the contact area of hydrocarbons, causing the biodegradation of these compounds. They are also environmentally friendly, non-toxic and highly stable [1,2].

Biosurfactants through fermentation are synthesized by yeast, bacteria and filamentous fungi and can also be extracted from plants. They can be applied in different industries, such as cosmetics, pharmaceuticals, food, petroleum and textiles, and can be used in the application of contaminated soils [3,4].

Soil is an essential environmental element that constitutes the ecosystem. It provides the basis for the productivity of plants and animals, in addition, it supports the survival and development of human beings. One of the main concerns related to soil is its contamination by the release of heavy metals through industrial and urban waste. Metal concentrations above limits affect the quality and microbiological balance of soils and can reduce soil fertility [5,6].

Some industrial processes are one of the main causes of heavy metal pollution, leading to their dispersion into terrestrial and aquatic ecosystems, causing environmental pollution. The gradual acclimatization of these soluble metal ions above their permitted limits and their accumulation in the food chain exert toxic and adverse effects on the environment, humans and animals. These heavy metal ions are persistent and can cause effects even at low concentrations [7].

With global industrialization, a large amount of heavy metals have been released into the oceans. Lead (Pb), mercury (Hg), arsenic (As), cadmium (Cd) and chromium (Cr) are the most problematic heavy metals that cause the greatest potential risk to humans and ecosystems due to their high toxicity [8,9].

Globally, there are 5 million sites with polluted soils, with areas covering approximately 500 million hectares of land; these areas are contaminated by heavy metals or metalloids, with current soil concentrations exceeding the geographic baseline or regulatory levels. It is estimated that pollution caused by heavy metals in soil has a global economic impact of more than 10 million dollars per year. This type of pollution is a major global issue because of growing concerns about the safety of agricultural products [10].

A study carried out by the Ministry of Environmental Protection (MEP) in China reported that the total area of arable land that is contaminated by heavy metals reached around 20 mha. It is estimated that more than 16% of total arable land and almost 20% of local recognized agricultural land had levels above the normal range for heavy metal accumulation. Furthermore, 13.7%, 2.8%, 1.8% and 1.1% of the land considered arable had slight, mild, moderate and heavy pollution levels, respectively [11].

Second to the Central Water Commission (CWC) of India, 42 rivers have at least two hazardous heavy metals beyond the permissible limit [12]. The Central Ground Water Board (CGWB) from India says that groundwater in more than 718 districts is affected due to the toxicity of heavy metals like Cd, Cr, As, Pb and Fe. This is a major problem considering that the heavy metals present in contaminated soils harm natural ecosystem services and, as a consequence, harm humans through the trophic chain [13,14,15].

The main concern about heavy metals is that these molecules bind to soil particles, due to their high interfacial tension and low solubility in water, making remediation of contaminated environments difficult. Biosurfactants can trap heavy metals and hydrocarbons and degrade them into products that can be used by the environment or even to be further degraded by soil microorganisms [16].

In other studies, *Candida bombicola* has demonstrated great potential for biosurfactant production and is widely used in several applications, such as in the food industry and for oil removal, but has not been reported in any previous study on the removal of heavy metals. In this sense, this work aimed to produce a low-cost biosurfactant for application in the remediation of environments contaminated by heavy metals.

## 2. Materials and Methods

### 2.1. Microorganism

The yeast tested as a biosurfactant producer was *Candida bombicola* URM3718 deposited in the Culture Collection of the Mycology Department of the Federal University of Pernambuco. Cultures were maintained in assay tubes containing Yeast Mold Agar (YMA), medium consisting of yeast extract (0.3%), malt extract (0.3%), D-glucose (1%), tryptone (0.5%) and agar (2%), under refrigeration at 5 °C.

### 2.2. Substrates of the Production Medium

Industrial waste was used as a substrate for the production of the biosurfactant. The corn steep liquor, residue from the manufacture of corn-based products used in the production medium, was obtained from Corn Products from Brazil, in the municipality of Cabo de Santo Agostinho, Pernambuco, Brazil. The molasses was obtained from the São José plant, located in the municipality of Igarassu, Pernambuco, Brazil. The residual frying oil was obtained from local restaurants in the city of Recife, Pernambuco, Brazil.

### 2.3. Inoculum Preparation

The yeast was transferred from a tube containing YMA to Erlenmeyer flasks containing 50 mL of Yeast Mold Broth (YMB) medium, consisting of yeast extract (0.3%), malt extract (0.3%), D-glucose (1%), tryptone (0.5.%) and incubated under shaking at 150 rpm at 28 °C for 24 h. After this period, dilutions and cell counting were performed in the Neubauer Chamber to obtain a final concentration of 10^4^ cells/mL.

### 2.4. Biosurfactant Production

The biosurfactant was produced by the yeast *C. bombicola* using industrial waste as low-cost substrates. The yeast was cultivated in medium containing distilled water supplemented with 5.0% molasses, 3.0% corn steep liquor and 2.5% residual frying oil. The initial pH of the medium was adjusted to 5.5 and then incubated with the cell suspension of 10^4^ cells/mL for 144 h in an orbital shaker at 200 rpm, according to previously established conditions [17].

### 2.5. Determination of Surface Tension and Critical Micelle Concentration

The surface tension of the cell-free metabolic liquid of the formulated biosurfactant was measured on a KSV Sigma 700 tensiometer (Finland) using the NUOY ring. Surface tension was measured by immersing the platinum ring in the metabolic liquid and recording the force required to pull it through the air–liquid interface. The critical micelle concentration (CMC) was determined by measuring the surface tension of dilutions of the isolated biosurfactant in distilled water for a constant surface tension value. The CMC value was obtained from the graph of surface tension and biosurfactant concentration and was expressed in g/L of biosurfactant [18].

### 2.6. Biosurfactant Isolation

The biosurfactant produced was extracted using a method developed in the laboratory that consisted of the use of an organic solvent, ethyl acetate. The extraction was performed in a 1:4 ratio of the volume of metabolic liquid and solvent, respectively. The process was repeated three times, and then the solvent was centrifuged for 15 min at 4500 rpm. Then, the organic phase was transferred to a separation funnel, discarding the possible aqueous phase evident due to washing of the sample using the addition of a saturated sodium chloride solution (NaCl). The solvent was dried with sodium sulfate and filtered. Finally, the organic phase was taken to a hot plate to evaporate the solvent and obtain the isolated biosurfactant.

### 2.7. Determination of the Emulsification Index

The emulsification index was determined using the method described by Cooper and Goldenberg [19], where 2 mL of a hydrocarbon was added to 2 mL of the cell-free metabolic liquid in an assay tube and vortexed for 2 min. Emulsion stability was determined after 24 h and the emulsification index was calculated by dividing the height of the emulsion by the total height of the mixture and multiplying by 100.

### 2.8. Stability Studies

The effect of adding different concentrations of NaCl on biosurfactant activity was investigated using cell-free metabolic liquid. Specific concentrations of NaCl (2–12%) were added and surface tension and emulsifying activity were determined. Cell-free metabolic liquid was also kept at constant temperature (0, 5, 70, 100 and 120 °C) and used for surface tension and emulsification measurement. The effect of pH on surface tension was evaluated after adjusting the metabolic liquid to pHs (2, 4, 6, 8, 10 and 12). All the tests were carried out independently; that is, separately.

### 2.9. Determination of Ionic Charge

The determination of the ionic charge of the biosurfactant was carried out using the Zeta Potentiometer with system Zeta -Meter 4.0 + ZM3-DG Direct Imaging (Zeta Meter, Inc., Harrisonburg, VA, USA).

### 2.10. Fourier-Transform Infrared Spectroscopy (FTIR)

The characterization of the chemical structure and components of the isolated biosurfactant sample were determined using a Fourier-transform infrared (FTIR) spectroscope (Spectrum 400, Perkin Elmer, Shelton, CT, USA) [3].

### 2.11. Nuclear Magnetic Resonance Spectroscopy (NMR)

The characterization of the chemical structure and components of the isolated biosurfactant sample were determined using nuclear magnetic resonance spectroscopy. The isolated and purified biosurfactant was dissolved in chloroform and the solutions were placed in a nuclear magnetic tube and analyzed [3].

### 2.12. Phytotoxicity Assay

The phytotoxicity of the biosurfactant was evaluated in a static test based on seed germination and root elongation of the lettuce (*Lactuca sativa)* according to the methodology described by Tiquia et al. [20]. The crude biosurfactant and isolated biosurfactant solutions were prepared with distilled water at concentrations of ½ CMC (0.25%, 1CMC (0.5%) and 2CMC (1.0%) The toxicity was determined in sterilized Petri dishes containing filter paper. Before the toxicity test, the seeds were treated with sodium hypochlorite. Ten seeds were inoculated into each Petri dish with 5 mL of the test solutions. After five days of incubation in the dark, seed germination and root elongation were determined as follows:

Relative seed germination (%) = (number of seeds germinated in the extract/number of seeds germinated in the control) × 100.

Relative root length (%) = (average root length in extract/average root length in control) × 100.

Germination index = [(% seed germination) × (% root growth)]/100.

### 2.13. Toxicity Assay Using Eisenia Fetida

The earthworm species *Eisenia fetida* was grown in the laboratory in soils rich in organic matter. The soil used for the escape test of 48 h was purchased from a construction store and consisted of a mixture of vegetable sand and washed sand in a 1:2 ratio, respectively. The soil passed by the defaunation process, where it was subjected to three cycles of freezing and thawing (48 h of freezing and 48 h of thawing), totaling 12 days for this process.

In addition to cell-free metabolic liquid, concentrations of ½ CMC (0.25%) and 1CMC (1.0%) were tested. The negative control was performed using distilled water and for the positive control boric acid was used. In total, 5 replicates of each tested condition were carried out, totaling 20 boxes containing 1 kg of soil, 500 g of control soil and 500 g corresponding to the soil with the pre-established solutions.

To start the test, earthworms were separated into Petri dishes, with 10 individuals for each box. The control soils and the solutions tested were separated by a plastic divider; posteriorly these plastic dividers were removed and the worms were placed in the center of the box to choose the soils, and finally the boxes were covered to prevent the worms from escaping.

After the period of 48 h incubation, the earthworms in the test soil and the control soil were observed for behavioral reactions of spiraling, changes in digging capacity, agitation and rejection of the contaminated soil [21]. The level of rejection of the test soil was indicated by applying the Test-t through the difference of individuals between test and control; a result *p* < 0.05 of the earthworms found in the control soil was considered significant [22].

### 2.14. Collection of Soil Contaminated with Heavy Metals

Samples of the soil were collected in the municipality of Belo Jardim, a region of automotive battery industries, located in the northern rural region of the State of Pernambuco [23].

### 2.15. Dynamic Treatment of Soils Contaminated by Heavy Metals

The soil washing process was carried out with the concentration of the biosurfactant solution (½ CMC, 1CMC and 2CMC) and the cell-free metabolic liquid. To select the best concentration of biosurfactant to remove the heavy metal, 5 g of the soil was placed in Erlenmeyer flasks with 125 mL capacity and 50 mL of the biosurfactant solution at different concentrations was added. The soil sample was simultaneously treated with distilled water in order to determine the removal of metals by physical mixing (control). The samples were incubated in a shaker with orbital rotation at 200 rpm for 24 h at 27 °C and were subsequently centrifuged at 5000 g for 10 min. The supernatant liquid was subjected to heavy metal quantification using the spectrometer ICP-OES Optima 7000 DV, PerkinElmer [23].

### 2.16. Static Treatment of Soils Contaminated by Heavy Metals

The soil contaminated with metals was placed in glass columns (57 cm in height and 3.0 cm diameter) and maintained at room temperature. The solutions used to move the contaminant adsorbed in the soil were as follows: distilled water as control; cell-free metabolic liquid; and concentrations of isolated biosurfactant (½ CMC, 1CMC and 2CMC). The soil sample was simultaneously treated with distilled water in order to determine the removal of metals by physical mixing (control) [24]. After washing the columns, the soil samples were subjected to heavy metal quantification using the spectrometer ICP-OES Optima 7000 DV spectrometer, PerkinElmer [25].

### 2.17. Kinetics of Heavy Metal Removal by Biosurfactant

Samples of 5.0 g of soil were added to 50 mL of the crude biosurfactant solution and shaken at 150 rpm for 0.5, 1, 3, 6, 12 and 24 h and then centrifuged at 5000 g for 15 min. After washing, the soil samples were subjected to heavy metal quantification using the spectrometer ICP-OES Optima 7000 DV, PerkinElmer [25].

### 2.18. Conductivity

The conductivity of the biosurfactant was measured on a conductivity meter Tec-4 MP. Solutions of the isolated biosurfactant (½ CMC, 1CMC and 2CMC) were added to solutions of a synthetic effluent prepared with lead nitrate and cadmium nitrate, separately, at a concentration of 500 mg/L in deionized water. The metal–biosurfactant precipitate was removed and the conductivity of the solution measured [26].

## 3. Results and Discussion

### 3.1. Biosurfactant Production and Yield

Surfactants of microbial origin, called biosurfactants, are compounds that play an important role in different industries and have a wide variety of applications; they can be used in pharmaceutical, cosmetic, cleaning and food areas. From an environmental point of view, biosurfactants are preferred due to their greater biodegradability characteristics and because they are obtained from natural sources. However, the processes involving the production of biosurfactants are still very expensive, causing a disadvantage compared to the production of synthetic surfactants in terms of costs [27].

Therefore, it is important and necessary to search for more profitable alternatives for this production. In this sense, fermentation was carried out using as components a low-cost medium formulated for the production of the biosurfactant by *C. bombicola* URM 3718. Production was carried out in a medium containing distilled water supplemented with 5.0% molasses, 3.0% corn steep liquor and 2.5% residual frying oil.

Corn steep liquor is a by-product resulting from the wet grinding of corn which contains a variety of aminoacids, inorganic salts and vitamins [28] and acts as a source of nitrogen in the production medium. Molasses is a by-product of the sugar industry and acts as a source of carbon in the production. The main components of molasses include sucrose (30–35%), fructose and glucose (10–25%), non-sugar compounds (2–3%), mineral and moisture content, and about 45–55% fermentable sugars. Furthermore, in contrast to refined sugars, small amounts of vitamins and several minerals are found in molasses [29]. Residual frying oil also constitutes a source of carbon and energy for the synthesis of biomolecules in production [30].

After 144 h of cultivation, the biosurfactant obtained from *C. bombicola* showed a capacity to reduce surface tension from 72 to 29 mN/m and a yield of 13 g/L, using ethyl acetate as a solvent. Measuring surface tension is one of the main methods frequently used to detect biological surfactants in culture media [31]. According to Akbari et al. [4], an effective biosurfactant is capable of reducing the surface tension of water from 72 to 35 mN/m.

According to Asgher et al. [32], the key to biosurfactant production is the optimization of the bioprocess, which consequently influences the total production yield. There are a variety of factors that influence the production of biosurfactants, with accessibility to carbon and nitrogen sources strongly contributing to production.

Lira et al. [33] produced biosurfactants using the yeast *C. guilliermondii* cultivated in a medium with 5.0% molasses, 5.0% corn steep liquor and 5.0% residual frying oil, and the surface tension of the water was reduced from 72 to 28.6 mN/m. Regarding the isolation of the biosurfactant, one of the conditions tested used ethyl acetate as the extraction solvent and the yield was 21 g/L.

Silva et al. [34], using a medium supplemented with 5.0% molasses, 5.0% residual soybean frying oil and 3.0% corn steep liquor in the production of biosurfactants using *C. bombicola*, observed a reduction in surface tension to 30.7 mN/m and the biosurfactant yield was 25 g/L, using ethyl acetate as extraction solvent.

Ribeiro, Guerra and Sarubbo [35] produced a biosurfactant using the yeast *Saccharomyces cerevisiae* in la ow-cost medium formulated in distilled water supplemented with 1.0% soybean oil residue and 1.0% corn steep liquor and observed that the surface tension of the culture medium was reduced from 57.7 to 26.6 mN/m. Furthermore, they used ethyl acetate as a biosurfactant extraction solvent and obtained a yield of 5.84 g/L.

Silva et al. [36] observed that after the production of a commercial biosurfactant using *Starmerella bombicola* in a medium containing 50 g/L of cottonseed oil, 25 g/L of glucose, 1 g/L of yeast extract, 0.5 g/L of KH_2_PO_4_, 0.5 g/L of MgSO_4_.7H_2_O and 0.3 g/L of NaNO_3_, surface tension was reduced to 32.3 mN/m and the biosurfactant isolated using ethyl acetate as an extraction solvent showed a yield of 32.5 g/L.

Santos et al. [37] carried out the fermentation of a biosurfactant using *C. lipolytica* in a medium formulated with distilled water supplemented with 4.0% molasses, 2.5% corn steep liquor and 2.5% used frying oil and observed a reduction in tension water surface from 70 to 29 mN/m, and after isolation of the biosurfactant using ethyl acetate as extraction solvent, a yield of 24 g/L was obtained.

Santos et al. [3] used the yeast *C. sphaerica* to produce a biosurfactant in a bioreactor, with a medium composed of distilled water supplemented with 9.0% peanut oil refinery residue and 9.0% corn steep liquor, and observed that the biosurfactant reduced the surface tension of the culture medium from 56 to 25.2 mN/m, and the biosurfactant yield was 10 g/L after its isolation using ethyl acetate as the extraction solvent.

### 3.2. Surface Tension and Critical Micelle Concentration

For a surfactant to be truly effective, the molecule must be amphipathic; that is, it must contain a mixture of hydrophobic and hydrophilic components so that it can act on both types of surfaces [38].

The biosurfactant produced by *C. bombicola* exhibited an excellent ability to reduce surface tension, taking into account that the water tension was reduced from 72 mN/m to 29 mN/m with increasing biosurfactant concentration to 0.5%; that is, indicating that the CMC was reached at this concentration.

The *C. bombicola* biosurfactant demonstrated a lower CMC compared to other biosurfactants produced *by Candida utilis* (0.6%) [39], *Saccharomyces cerevisiae* (0.8%) [35] and *Yarrowia lipolytica* (1.2%) [40].

According to Kumar et al. [41], biosurfactants have been applied to reduce surface and/or interfacial tension between the solution and the surface at air/water or oil/water interfaces. The addition of a surfactant to an air/water or oil/water system causes a reduction in surface tension to a point where they form structures such as micelles, vesicles and bilayers. This critical point is known as critical micellar concentration (CMC). The low molecular weight of surfactants produced and synthesized by microorganisms has a great capacity to reduce surface tension. On the other hand, the high molecular weight is related to the ability to form a stable emulsion. According to Twigg et al. [42], CMC is the concentration of a surfactant compound in solution at the moment micelles are formed.

### 3.3. Biosurfactant Stability

Bioemulsifiers are a group of molecules that can be used to replace biosurfactants. These molecules have an active surface and an emulsion between hydrocarbons and water mixtures that quickly reduce surface tension [43]. Determination of the emulsification index is a method used to identify emulsifiers that are characterized by their excellent emulsion stabilization capacity. The use of bioemulsifiers in various industrial areas depends on their stability in different temperature, pH and salinity conditions [44].

A biosurfactant needs to maintain its surfactant characteristics under any environmental conditions [45]. Environmental factors such as pH, temperature and salinity affect the activity and stability of a biosurfactant; therefore, it is of great importance to study the influence of these variables [25].

The stability of the biosurfactant produced by *C. bombicola* was evaluated using cell-free metabolic liquid after 144 h of fermentation against different variables. Different pH values (2, 4, 6, 8, 10 and 12), temperature (0, 5, 70, 100 and 120 °C) and NaCl concentrations (2, 4, 6, 8, 10 and 12%) were tested for surface tension and emulsification using engine oil.

According to Table 1, the surface tension reduction capacity remained stable and varied from 30 to 35 mN/m in relation to pH, 30 to 31 mN/m regarding NaCl concentrations and 29 to 31 mN/m at different temperatures. When subjected to emulsification activity, the formulated biosurfactant remained stable under all temperature variations, reaching 100% emulsification of the engine oil; it varied from 86% to 95% in relation to different pHs and 84 to 89% in relation to NaCl concentrations (Table 2).

Rocha Junior et al. [25] used *C. tropicalis* in the production of a biosurfactant in a growth medium containing 4.0% corn steep liquor, 2.5% molasses and 2.5% canola oil and obtained similar results in relation to emulsification stability. Emulsification tests demonstrated a high degree of affinity between the metabolic liquid and the engine oil, and the constant stability of the emulsification was around 92% in the tested temperature and pH ranges. Regarding salinity, the emulsification capacity was maintained only in the presence of 2% NaCl (compatible with the saline concentration found in the ocean); however, with NaCl concentrations greater than 4%, emulsification decreased.

### 3.4. Ionic Charge of the Biosurfactant

The biosurfactant produced by *C. bombicola* showed a negative charge in the hydrophilic region after analysis in a zeta potentiometer, with +61.4 ZPmV, 160.6 µS/cm at 25.7 °C, indicating that it is an anionic surfactant. Compared to the literature, other biosurfactants produced by *Candida* species also showed an anionic character when subjected to the same test [25,46].

### 3.5. Fourier-Transform Infrared Spectroscopy (FTIR) and Nuclear Magnetic Resonance (NMR)

**Figure 1:**
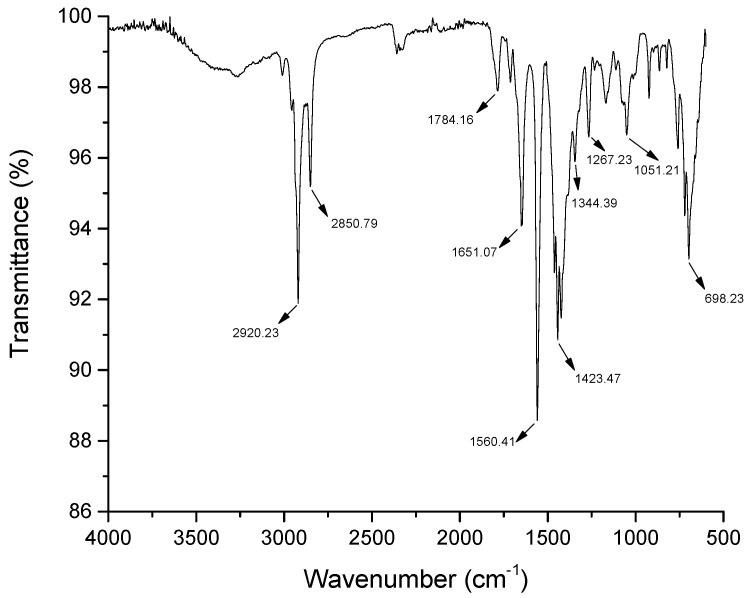
FTIR spectrum of the isolated biosurfactant produced by *C. bombicola* in distilled water supplemented with 5.0% molasses, 3.0% corn steep liquor and 2.5% residual frying oil.

According to the infrared spectrum (Figure 1), the biosurfactant shows stretches located at 2920 cm^−1^ and 2850 cm^−1^, attributed to the saturated carbons present in the molecule (CH), which are responsible for the hydrophobic fraction of the structure. The signal at 1560 cm^−1^ can be attributed to a carbonyl (C=O) which is probably associated with a hydroxyl (OH), forming the carboxylic acid function.

**Figure 2:**
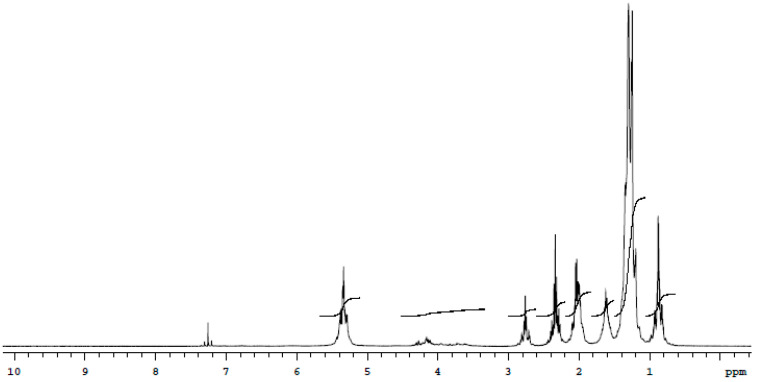
^1^H MNR spectrum (CD_3_OD, 300 MHz) of the isolated biosurfactant of *C. bombicola* in distilled water supplemented with 5.0% molasses, 3.0% corn steep liquor and 2.5% residual frying oil.

The NMR of the ^1^H analysis of the semi-purified biosurfactant reveals the presence of the methyl functional group in the region between 0 and 1 ppm. The signals between 1 and 3 ppm are responsible for the hydrogens linked to the saturated aliphatic carbon chain. This sample can be a mixture of some fatty acids metabolized by the microorganism. The regions located between 2 and 3 ppm can be attributed to the signals of hydrogens bonded to carbons close to the more electrophilic functional groups, such as double bonds. The signal present in the region between 3.4 and 4.6 ppm could be interpreted as a possible presence of hydroxyls in the structure of the molecule; however, as this signal did not appear in the ^13^C NMR, we can infer that it is an aqueous contamination of the sample sent. The signals between 5 and 5.8 ppm are derived from the hydrogens present on the carbons of the double bond (Figure 2).

**Figure 3:**
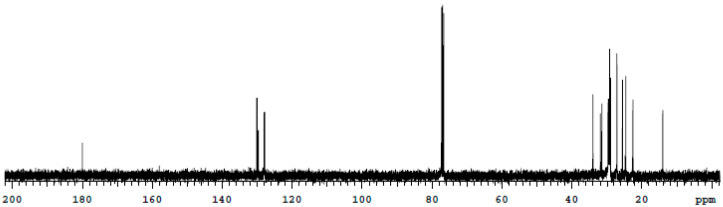
C MNR spectrum of the biosurfactant produced by *C. bombicola* in distilled water supplemented with 5.0% molasses, 3.0% corn steep liquor and 2.5% residual frying oil.

In the ^13^C NMR spectrum, we can find the signals of saturated aliphatic carbons in the region between 0 and 40 ppm and double bonds can be observed in the signal between 120 and 140 ppm and the characteristic signal that highlights the presence of the carboxylic acid function at the point 180 ppm. The signal between 70 and 80 ppm is attributed to the solvent (deuterated chloroform). There is no presence of signals in the region located between 40 and 70 ppm indicating the absence of hydroxyls in the structure, suggesting that the biosurfactant produced is a possible fatty acid of the straight chain and unsaturated-type resulting from the microorganism metabolism (Figure 3).

### 3.6. Phytotoxicity Assay

Phytotoxicity has a negative impact on plant growth or fitness and may be associated with cellular dysfunction [47]. The toxicity of the *C. bombicola* biosurfactant was evaluated using the plant species *Lactuca sativa*. The results demonstrate that the different biosurfactant solution treatments did not cause an inhibitory effect on seed germination and root growth. Germination rates varied by 51, 78 and 53% for solutions of ½ CMC, 1CMC and 2CMC, respectively.

Results presented by Santos et al. [48] indicated that their germination occurred even in the presence of high concentrations of the isolated biosurfactant.

Felix et al. [49] carried out a phytotoxicity test with *Lactuca sativa* of the biosurfactant produced by *Bacillus subtilis* and took into account root growth and the number of germinated seeds and observed that the biosurfactant produced did not influence root elongation and seed germination rate, even though the germination rate was 78% at concentrations of 500 mg L^−1^.

### 3.7. Toxicity Assays Using Eisenia Fetida

Earthworms are common soil macrofauna organisms and play a fundamental role in soil structure, nutrient cycling and microbial composition [50]. Furthermore, earthworms can be used as biological vectors to promote soil quality in the restoration of degraded environments [51].

Due to the displacement and ingestion of contaminated soil, earthworms come into contact with pollutants that reach the soil and remain adsorbed in mineral particles, organic matter and in the soil solution. In this way, they expose themselves to and absorb contaminants from the soil through direct contact and passage through the cuticle, causing them to become poisoned, die or survive, incorporating and even bioaccumulating these pollutants in their tissues. The potential of these organisms that are at lower levels of the trophic chain has been widely studied because due to their ecological niche and important trophic position, they serve as food for several animals and as a route for the transfer of biomagnification of contaminants throughout the trophic web, making earthworms excellent bioindicators of ecotoxicity of chemical substances in the soil as it indicates potential bioaccumulation throughout the chain [21].

*Eisenia fetida* is a species of oligochaete annelid that has been widely used as a test organism in acute and subchronic toxicity tests in contaminated soil to evaluate the effect of substances due to its sensitivity to chemical agents, wide availability as it is a species with great reproduction capacity and ease of growth under laboratory conditions. It is a sensitive bioindicator and offers numerous possibilities for studies of environmental contamination by pesticides, metals, petroleum derivatives, antibiotics, veterinary products and several other pollutants [21,22,52].

After a period of 48 h, the earthworms were found in all the biosurfactant conditions tested (cell-free metabolic liquid, ½ CMC and 1CMC) and in the control soils, demonstrating that they did not make a distinction in the choice of soils. No apparent adverse effects were found; they moved normally and demonstrated normal behavior. On the other hand, the earthworms used in the positive control test (boric acid) mostly fled to the control soil and moved more slowly. According to the results obtained, it was found that the test soils with concentrations of biosurfactants produced by *C. bombicola* did not cause toxicity to earthworms, considering the *p*-value > 0.05. In this sense, the biosurfactant can be used for bioremediation of environments contaminated by heavy metals and hydrophobic compounds without causing environmental damage.

**Figure 4:**
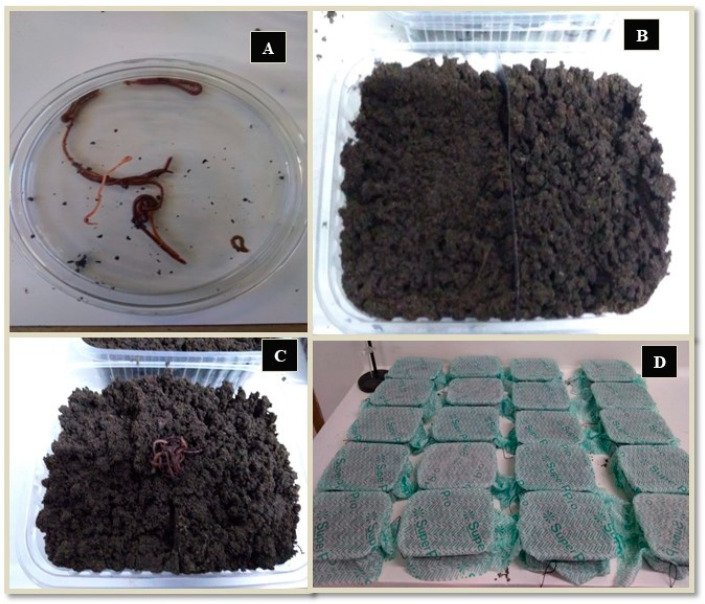
Ecotoxicological test using *Eisenia fetida* as test organism. (**A**) Earthworms separated into Petri dishes; (**B**) control soil and soil with solutions tested separated by a plastic divider; (**C**) plastic divider removed and the worms were placed in the center of the box; (**D**) boxes covered to prevent the worms from escaping.

Soroldoni et al. [53] studied acute and chronic toxicity in *Eisenia andrei* exposed to soil contaminated with 5% used oil, where biostimulation and bioaugmentation adding mature and unripe compounds from municipal solid waste were used as bioremediation strategies. It was found that even after bioremediation, toxic effects remained in the bioremediated soils, probably due to remaining hydrocarbons and/or hydrocarbon biodegradation products, causing high mortality rates in the earthworms used in the ecotoxicological test and anatomical deformations in the surviving specimens, demonstrating the sensitivity of earthworms to toxic contaminants. Figure 4 shows ecotoxicological tests using *Eisenia fetida* as test organism. Earthworms were separated into Petri dishes (A); control soils and the soils with solutions tested were separated by a plastic divider (B); the plastic divider was removed and the worms were placed in the center of the box to choose the soils (C); boxes were covered to prevent the worms from escaping (D).

### 3.8. Dynamic Treatment of Soil Contaminated by Heavy Metals

Biosurfactants are biologically active compounds that have been successfully used in the detoxification and/or removal of toxic heavy metals. Biosurfactants have several advantages in the remediation of heavy metals due to their small size, low toxicity, high biodegradability and activity at extreme pH and temperature levels. As they present a wide variety of chemical structures, a wide spectrum of metal selectivity and binding capacity, they provide greater removal capacity. Biosurfactants have a strong affinity for heavy metals, resulting in the formation of a biosurfactant–metal complex [54,55,56,57].

The low-cost biosurfactant produced by *C. bombicola* has been tested for its ability to remove heavy metals from soil samples. Solutions of the isolated biosurfactant at different concentrations were tested and the removal of metals using cell-free metabolic liquid was also evaluated, while distilled water was used as a control (Table 3).

According to the results obtained, it was possible to observe that the biosurfactant produced by *C. bombicola* achieved excellent heavy metal removal efficiency through the dynamic test. The data demonstrated that iron was the metal most removed, with values ranging between 78 and 88%, followed by zinc with 65 to 71% and lead with values from 25 to 48%. The cell-free metabolic liquid was able to significantly remove 48, 71 and 88% of lead, zinc and iron from the soil, respectively, and was the treatment tested with the highest percentage of removal, indicating the possibility of using the biosurfcattant without the purification. According to Almeida et al. [58], the purification of a biotechnological product represents around 60% of the total production cost, and this cost can be avoided by using the biosurfactant in raw form.

Rocha Junior et al. [25], Luna, Rufino and Sabubbo [23] and Sarubbo et al. [59] also carried out studies with different microorganisms and applied the biosurfactant produced to remove heavy metals and achieved successful results, demonstrating that biosurfactants produced by different species of the *Candida* genus are efficient in removing heavy metals contained in soils and effluents.

Rocha Junior et al. [25] evaluated the ability of biosurfactants produced by *C. tropicalis* to remove heavy metals in contaminated sand, and the removal rates for crude biosurfactant treatment were 60, 55 and 10% of Cu, Zn and Pb, respectively. The isolated biosurfactant also showed efficiency in removing Zn (35%), Cu (80%) and Pb (15%).

Luna, Rufino and Sabubbo [23] tested the efficiency of biosurfactants produced by *C. sphaerica* in removing heavy metals from contaminated soil and observed that the crude biosurfactant was able to remove 95, 90 and 79% of Fe, Zn and Pb, respectively. Removals of 89, 87 and 70% were obtained for Fe, Zn and Pb, respectively, when using the 2.5% biosurfactant solution.

Sarubbo et al. [59] applied the biosurfactant produced by *C. guilliermondii* in the removal of metals and obtained a percentage of 98, 89 and 89% removal for Zn, Fe and Pb, respectively, using the crude biosurfactant. Removals of 99, 96 and 93% were obtained for Fe, Pb and Zn, respectively, when using the 0.8% biosurfactant solution.

### 3.9. Static Treatment of Soil Contaminated by Heavy Metals

Considering the possibility of the in situ soil treatment, the biosurfactant *C. bombicola* was tested in packed columns containing contaminated soil. The removal rates demonstrated satisfactory efficiency and were slightly lower than those achieved in the dynamic test (Table 4). The results obtained demonstrate that the treatment of soils with a concentration of 1% biosurfactant achieved a greater capacity to remove metals with 40, 60 and 65% for Pb, Zn and Fe, respectively.

### 3.10. Kinetics of Heavy Metal Removal by Biosurfactant

**Figure 5:**
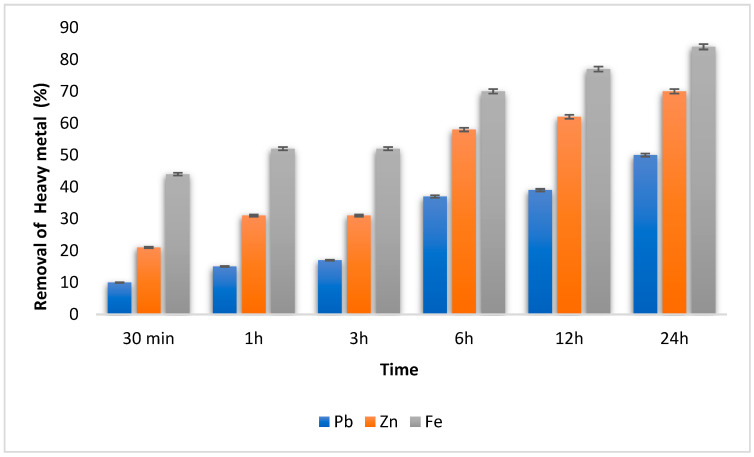
Removal of heavy metals by cell-free *C. bombicola* over time.

The kinetics of heavy metal removal were studied over a period of 24 h with the objective of reducing the biosurfactant action time and, consequently, the final cost of applying the bioproduct (Figure 5). The kinetic experiment showed that there was an equilibrium reached in the first 30 min of the test, accompanied by an increasing and constant percentage that reached 50, 70 and 85% for Pb, Zn and Fe, respectively, metal removal at the end of 24 h. According to Narimannejad, Zhang and Lye [60], adsorption dynamics, especially kinetic models, have been used to trace the associated mechanisms and determine the adsorption rate of heavy metals in soil.

### 3.11. Removal of Heavy Metals Contained in Synthetic Effluent by Biosurfactant

Conductivity measurements are routinely used in many industrial and environmental applications as a quick, efficient and low-cost way of measuring the ionic character of a solution [25].

The conductivity of solutions containing the heavy metals cadmium (Cd) and lead (Pb) was reduced when the biosurfactant was added to the metal solutions at a concentration of ½ CMC due to the precipitation of the metals positively charged after being captured by the biosurfactant, reducing the amount of metal ions available in the metal solution and, consequently, the conductivity values of solution (Table 5).

Biosurfactants are highly attracted to heavy metals, leading to the formation of a stable biosurfactant–metal complex. This occurs due to the diversity of structures, broad spectrum of metal selectivity and binding capacity, leading to greater removal capacity [55,56,61].

According to Ayangbenro and Babalola [62] in the metal removal mechanism, biosurfactants act as a link between the fluid interface due to their amphiphilic nature, causing a reduction in surface tension. The decrease in water surface tension causes an increase in the mobilization of metal from the unsaturated soil, allowing its removal. In the mechanism of metal extraction through the microbial surfactant, ion exchange, counter bonding and precipitation occur. In ion exchange, the negatively charged anionic biosurfactant bonds with positively charged metal cations to form a stronger bond than the bond generated between the metal ion and the soil. During counter-binding, the polar portions of the surfactant micelles bind to the metal ions, leaving them soluble in water; thus, the recovery of the metal is carried out by washing. In the end, the metal–biosurfactant complex forms a strong bond where washing with water removes the complex from the soil matrix.

The conductivity of the biosurfactant solution increased with increasing concentration, reaching 407 and 392 µS/cm, at twice the CMC (2CMC), respectively. The increase in conductivity with increasing concentration of the biosurfactant solution is due to the anionic nature of the surfactant.

The results obtained also demonstrated the efficiency of the biosurfactant at the lowest concentration tested (½ CMC since a gradual increase in the conductivity of metal solutions was observed after the addition of biosurfactant solutions at higher concentrations (CMC and 2CMC), showing that the conductivity values increased again due to the ionic charge of the biosurfactant itself.

Tang et al. [63] observed that although it results in high electrical conductivity during the electrokinetic treatment coupled to a biosurfactant, it is possible that a combination of rhamnolipids, sophorolipids and saponin could promote the removal of heavy metals. In particular, the hydroxyl and carboxyl functional groups of biosurfactants react with heavy metals to form metallic compounds, migrating towards the cathode chamber during electrokinetic treatment.

## 4. Conclusions

The results demonstrate that the anionic biosurfactant obtained from *C. bombicola* is a surface-active agent with excellent capacity to reduce surface tension and is promising for use as a bioremediation technology in washing soils contaminated by heavy metals, presenting excellent removal efficiency. Under dynamic conditions, the crude and isolated biosurfactants demonstrated potential in removing Fe, Zn and Pb with percentages between 70 and 88%, with the highest Pb removal being 48%. The highest percentage of removal was obtained with the cell-free metabolic liquid, which was able to remove 48, 71 and 88% of Pb, Zn and Fe from the soil, respectively. Tests carried out in packed columns also confirmed the biosurfactant’s ability to remove Fe, Zn and Pb between 40 and 65%. The removal kinetics demonstrated an increasing percentage, achieving greater removal efficiency at the end of 24 h. The biosurfactant was also able to significantly reduce the electrical conductivity of solutions containing heavy metals. Furthermore, the biosurfactant produced presented characteristics such as biodegradability, chelating activity and stability in adverse conditions. The biosurfactant did not demonstrate toxicity when subjected to tests, demonstrating that it can be used in the environment without compromising the quality of soil, water, fauna and flora. However, it is important that more in-depth research be carried out on other parameters that will contribute to the effective success of this remediation technique.

## Figures and Tables

**Table 1:** Stability of the surface tension of the biosurfactant produced by *C. bombicola* in distilled water supplemented with 5.0% molasses, 3.0% corn steep liquor and 2.5% residual frying oil as substrates against temperature variations, pH and in the presence of NaCl.

NaCl (%)	Surface Tension (mN/m)	Temperature (°C)	Surface Tension (mN/m)	pH	Surface Tension (mN/m)
0	29 ± 1.3	0	30 ± 1.1	0	29 ± 1.3
2	30 ± 1.3	5	30 ± 1.2	2	35 ± 1.1
4	30 ± 1.8	70	29 ± 2.0	4	34 ± 1.2
6	30 ± 1.2	100	31 ± 1.2	6	30 ± 1.2
8	30 ± 1.4	120	29 ± 1.3	8	28 ± 1.3
10	31 ± 1.1			10	31 ± 1.2
12	31 ± 1.2			12	33 ± 1.2

Experiments were performed in duplicate and the results represent the mean ± standard deviation of two independent experiments.

**Table 2:** Stability of engine oil emulsification by biosurfactant produced by *C. bombicola* in distilled water supplemented with 5.0% molasses, 3.0% corn steep liquor and 2.5% residual frying oil as substrates against variations in temperature, pH and in the presence of NaCl.

NaCl (%)	Engine Oil Emulsification (%)	Temperature (°C)	Engine Oil Emulsification (%)	pH	Engine Oil Emulsification (%)
0	90 ± 1.2	0	100 ± 1.1	0	90 ± 1.2
2	89 ± 1.3	5	100 ± 1.3	2	86 ± 1.1
4	89 ± 1.8	70	100 ± 1.2	4	90 ± 1.7
6	89 ± 1.2	100	100 ± 1.2	6	92 ± 1.2
8	89 ± 1.4	120	100 ± 1.3	8	89 ± 1.5
10	84 ± 1.1			10	94 ± 1.2
12	87 ± 1.2			12	95 ± 1.2

Experiments were performed in duplicate and the results represent the mean ± standard deviation of two independent experiments.

**Table 3:** Removal of heavy metals, lead (Pb), iron (Fe) and zinc (Zn) contained in soil contaminated by treatments used in dynamic testing.

Treatments	Removal (%)		
	Pb	Zn	Fe
Distilled water (control)	10 ± 1.0	15 ± 1.1	12 ± 1.3
Cell-free metabolic fluid	48 ± 1.2	71 ± 1.5	88 ± 1.4
0.25% biosurfactant solution (½ CMC)	25 ± 1.4	65 ± 1.2	78 ± 1.4
0.5% biosurfactant solution (1CMC)	33 ± 2.1	68 ± 1.7	84 ± 1.2
1% biosurfactant solution (2CMC)	45 ± 1.4	70 ± 1.2	80 ± 2.0

**Table 4:** Removal of heavy metals, lead (Pb), iron (Fe) and zinc (Zn) contained in soil contaminated by treatments used in static testing.

Treatments	Removal (%)		
	Pb	Zn	Fe
Distilled water (control)	5 ± 1.0	11 ± 1.1	13 ± 1.3
Cell-free metabolic fluid	37 ± 1.2	58 ± 1.2	60 ± 1.3
0.25% biosurfactant solution (½ CMC)	30 ± 1.1	51 ± 1.2	57 ± 1.6
0.5% biosurfactant solution (1CMC)	35 ± 1.3	56 ± 1.7	61 ± 1.3
1% biosurfactant solution (2CMC)	40 ± 1.1	60 ± 1.2	65 ± 1.0

**Table 5:** Conductivity before and after the addition of biosurfactant, in different concentrations, in metallic solutions containing lead (Pb) and cadmium (Cd).

Heavy Metal	Conductivity of Metallic Solutions (µS/Cm)	Conductivity of the Solution after Addition of the Biosurfactant		
		½ CMC	1CMC	2CMC
Pb	670.3	382 ± 1.2	387 ± 1.1	407 ± 1.6
Cd	512.4	360 ± 1.4	366 ± 1.2	392 ± 1.1

## Data Availability

Data are contained within the article.
